# Psychological correlates of self-reported functional limitation in patients with ankylosing spondylitis

**DOI:** 10.1186/ar2874

**Published:** 2009-12-07

**Authors:** Tamar F Brionez, Shervin Assassi, John D Reveille, Thomas J Learch, Laura Diekman, Michael M Ward, John C Davis, Michael H Weisman, Perry Nicassio

**Affiliations:** 1 Department of Medicine, Division of Rheumatology, University of Texas-Houston, 6431 Fannin, Houston, Texas 77030, USA; 2 Department of Medicine, Division of Rheumatology, Cedars-Sinai Medical Center, 8700 Beverly Blvd., Los Angeles, California 90048, USA; 3 Department of Medicine, Division of Rheumatology, NIAMS-NIH, 1 AIMS Circle, Bethesda, Maryland 20892, USA; 4 Department of Medicine, Division of Rheumatology, University of California-San Francisco, 505 Parnassus Avenue, San Francisco, California 94122, USA; 5 Department of Psychiatry, University of California-Los Angeles, 300 Medical Plaza, Los Angeles, California 90095, USA

## Abstract

**Introduction:**

Functional status is an integral component of health-related quality of life in patients with ankylosing spondylitis (AS). The purpose of this study was to investigate the role of psychological variables in self-reported functional limitation in patients with AS, while controlling for demographic and medical variables.

**Methods:**

294 AS patients meeting modified New York Criteria completed psychological measures evaluating depression, resilience, active and passive coping, internality and helplessness at the baseline visit. Demographic, clinical, and radiologic data were also collected. Univariate and multivariate analyses were completed to determine the strength of correlation of psychological variables with functional limitation, as measured by the Bath AS Functional Index (BASFI).

**Results:**

In the multivariate regression analysis, the psychological variables contributed significantly to the variance in BASFI scores, adding an additional 24% to the overall R-square beyond that accounted by demographic and medical variables (R-square 32%), resulting in a final R-square of 56%. Specifically, arthritis helplessness, depression and passive coping beside age, ESR and the Bath AS Radiograph Index accounted for a significant portion of the variance in BASFI scores in the final model.

**Conclusions:**

Arthritis helplessness, depression, and passive coping accounted for significant variability in self-reported functional limitation beyond demographic and clinical variables in patients with AS. Psychological health should be examined and accounted for when assessing functional status in the AS patients.

## Introduction

With the improvement in prognosis due to advances in treatment, there is greater focus now on the patient's perspective on disease activity and quality of life [1-3]. Functional status is an integral component of health-related quality of life, and is important to patients with ankylosing spondylitis (AS) [4]. Poor functional status is correlated with work disability and increased medical costs in AS [4-8], lending to the increasing body of research examining the major determinants of functional limitations in the AS population.

Markers of disease activity (erythrocyte sedimentation rate (ESR), C-reactive protein, radiograph severity, disease duration) and socio-demographic variables do not fully account for the variability in patients' functional limitations, suggesting that additional factors, such as psychosocial variables, might play an important role [9]. Radiographic severity, higher disease activity scores, smoking [10], advanced age, lower education level, longer disease duration, presence of co-morbid medical conditions, and female gender are all associated with greater limitation; however, few studies have investigated the contribution of psychological factors to functional impairment in AS, and none have weighed the relative impact of psychological variables compared with these other factors [11-14].

Two prior studies, examining the role of psychological factors in AS functional limitation, found functional disability, measured by the Bath AS Functional Index (BASFI), to be associated with higher depression scores and lower internality scores in a UK AS population, and depression to be highly correlated with work disability and unemployment in an Argentinean AS population [15,16]. However, these studies examined only a limited number of potential variables and did not use multivariate analyses to account for the confounding effect of multiple baseline variables when they are examined simultaneously.

As emotional problems are present in approximately one-third of patients with inflammatory rheumatic conditions, ranging from 20% to 31% of patients with AS, and the correlation of functional limitation and depression is well documented in chronic arthritides such as rheumatoid arthritis (RA), it is important to investigate the contribution of psychological factors to functional limitation in patients with AS [13,17-19].

The purpose of this study is to investigate the correlation of psychological variables, independent of important demographic and biologic factors, on functional limitation, as measured by the BASFI, in a large AS cohort.

## Materials and methods

### Patients

Study participants were enrolled in the Prospective Study of Outcomes in Ankylosing Spondylitis (PSOAS), a longitudinal study of AS patients recruited from four US study sites: Cedars-Sinai Medical Center, Los Angeles, CA; the National Institutes of Health, Bethesda, MD; the University of Texas Health Science Center at Houston, Houston, TX; and the University of California, San Francisco, CA. Recruitment occurred via three avenues: academic rheumatology clinics at the above US study sites, internet advertisements, and patients enrolled in prior clinical studies at the above sites were invited to participate. All patients met the Modified New York Criteria for AS [15,20]. All the 294 enrolled patients in the longitudinal PSOAS study were included in the current study. This study was conducted in compliance with the Helsinki Declaration to protect human subjects and was approved by the Institutional Review Boards of the participating sites. All participating patients gave written informed consent according to the Institutional Review Boards specifications.

### Study design

Baseline assessments completed at each academic study site included medical history, socio-demographic information, psychological status, as well as radiographs of the pelvis, lumbar spine, and cervical spine. The majority of radiographs (58%) were completed at the time of the cross-sectional survey at the enrollment; the time between enrollment and radiographic examination was generally short (mean: 63 days).

### Primary outcome

The primary outcome used was the BASFI, with a score range of 0 mm to 100 mm. The BASFI is a self-report 10-item questionnaire developed by a team of medical professionals and patients. The first eight questions cover function in AS, while the final two explore the patient's ability to cope with the happenings of everyday life. Each question is answered on a 100 mm visual analogue scale (VAS), from none (0 mm) to very severe (100 mm), and the average determines the final BASFI score (0 to 100). Lower scores indicate a better functional status [21].

### Independent variables

Our database includes variables from the following domains: socioeconomic-demographic, immunologic, genetic, psychological, and clinical. We only describe the variables included in the final analyses below.

Socio-demographic information included age (at cross-sectional study baseline), education level (≤ 12 years, 13 to 15 years, 16 years, and > 16 years), ethnicity (white vs. other), current employment and student status as binary variables.

Medical variables consisted of an inflammatory marker (ESR), current tobacco use, number of co-morbid medical conditions (0 to 4 or greater), current non-steroidal anti-inflammatory drug (NSAID) use and biologic therapy (yes vs. no), disease duration (at time of cross-sectional survey), and radiographic score. We also investigated the relation between exercise habits and BASFI. For this purpose, the frequency of general exercise per week, performance of back stretching or strengthening exercises (yes vs. no) and physical therapy for treatment of AS in the past four months (yes vs. no) were investigated as independent variables. Each participant also had baseline radiographs of the pelvis (anterior-posterior), lumbar spine (anterior-posterior and lateral) and cervical spine (lateral), which were scored using the Bath AS Radiographic Index Global (BASRI-global) by a single musculoskeletal radiologist (TJL) at study entry. The BASRI-global is a validated method to score radiographic severity in patients with AS, including both hip and spine scores, with a score range of 1.5 to 16 [22].

We have observed in a previous study that the self-reported disease activity in AS, as measured by the Bath AS Disease Activity Index (BASDAI) [23], highly correlates with the psychometric variables (manuscript in review). Therefore, we did not include BASDAI as one of the independent variables in our analysis and relied more on objective surrogates of disease activity such as ESR because the inclusion of perceived disease activity might have masked the relation between the psychometric factors and the self-reported disease disability.

Six psychological variables were measured: active and passive coping, depression, resilience coping, helplessness and internality. The Vanderbilt Pain Management Inventory (VPMI) is an 18-item self-report questionnaire that assesses the frequency of utilization of coping strategies in patients with chronic pain when their pain is at a moderate level of intensity or greater. The VPMI has two validated subscales: active coping and passive coping [24]. The Patient Health Questionnaire (PHQ-9) is a brief, valid, nine-item self-report instrument that has primarily been designed for detecting depressive disorders in primary care settings. An advantage of the PHQ-9 is that its items are based on the actual criteria upon which the diagnosis of *Diagnostic and Statistical Manual of Mental Disorders*-IV depressive diagnosis is made [25-27] and do not overlap with medical symptoms as extensively as many other depression measures. Score can range from 0 to 27, as each of the nine items can be scored from 0 (not at all) to 3 (nearly every day). The Brief Resilient Coping Scale (BRCS) is a four-item self-report measure that measures patients' ability to feel challenged by, and cope adaptively, with adversity. BRCS scores can range from 0 to 20, with higher scores indicating higher resilience [28]. The Arthritis Helplessness Index (AHI) is a 15-item self-report questionnaire designed to measure patient's perceptions of loss of control in association with their chronic arthritis [29]. We used the two subscales, internality (seven items) and helplessness (five items), which reflect separate constructs and have been found to have greater reliability and validity than the total AHI score [30]. Arthritis internality assesses patients' beliefs that their own behavior can control their arthritis, while arthritis helplessness assesses patients' perceptions of helplessness in coping with their chronic arthritic condition. The two subscales are inversely related to each other, but reflect largely independent beliefs about the controllability of arthritis [30].

### Statistical analysis

We conducted the data analysis in four steps. First, descriptive statistics were computed on our study cohort (Table 1). Second, we completed univariate linear regression analyses to evaluate which variables were associated with the BASFI (Table 2). Then, we examined the association of demographic, medical, and psychological factors with the BASFI using hierarchal regression modeling (Table 3). In order to analyze the contribution of these groups of variables to BASFI, we entered the variables in successive conceptual blocks: (1) demographic variables, (2) medical variables, (3) psychological measures. This order of entry tested the proposition that psychological factors would contribute unique variance to AS functional limitation independently of demographic and medical variables. Subsequently, a final model was established using a forward hierarchical variable selection strategy. This approach was chosen to decrease the effect of multicolinearity in our analysis. Initially we entered all variables into the model. Then, the number of independent variables was reduced to those that changed the R square of the entire model by 2% or more. Those variables were entered into the final model (Table 4).

**Table 1:** Sample characteristics

Demographic variables:	
Mean age, SD, years	45.1 (14.40)
Education level	
≤12 years, n, %	34 (11.6%)
13-15 years, n, %	81 (27.6%)
16 years, n, %	77 (26.2%)
> 16 years, n, %	102 (34.7%)
Gender, n, male, %	197 (68.2)
Ethnicity, n, white, %	241 (82.0)
Number employed, %	192 (65.5)
Number student, %	26 (8.9)
Married, n, yes, %	153 (55.8)
	
**Medical variables:**	

Current tobacco use, n, %	32 (11.0)
Mean number of medical co-morbidities (0-4), SD	2.0 (1.34)
Current NSAID use, n, %	136 (46.6)
Current biologic therapy, n, %	132 (45.2)
Mean erythrocyte sedimentation rate mm/hr, SD	14.9 (16.0)
Mean disease duration, SD, years	21.2 (13.85)
Mean Bath AS Radiographic Index (BASRI) score (1.5-16), SD	6.5 (4.27)
Mean frequency of exercise/week, SD	3.12 (2.34)
Performance of back stretching or strengthening exercises, n, %	188 (63.9)
Physical therapy in the past 4 months, n, %	25 (8.5)
	
**Psychological variables:**	

Mean resilience coping (BRCS) score (0-20), SD	16.1 (3.33)
Mean arthritis internality score (6-36), SD	25.7 (5.94)
Mean arthritis helplessness score (5-25), SD	12.4 (4.41)
Mean sepression (PHQ-9) score (0-27), SD	5.1 (5.01)
Mean active coping score (7-35), SD	22.7 (5.22)
Mean passive coping score (11-55), SD	25.6 (7.45)

n = 294. PHQ = Patient Health Questionnaire; SD = standard deviation.

**Table 2:** Univariate analyses of demographic, medical and psychological variables in relationship to BASFI

Independent variable	Mean difference in BASFI	95% confidence interval	*P *value
Age (at baseline visit)	0.48	0.28-0.68	**< 0.001**
Education	-3.07	-5.36--0.78	**0.009**
Gender (male)	-3.18	-9.48-3.12	0.322
Ethnicity (white)	-1.17	-8.73-6.39	0.761
Employment (yes)	-16.41	-22.27--10.54	**< 0.001**
Student (yes)	0.75	-9.49-10.99	0.885
Married (yes)	3.31	-2.76-9.38	0.284
Current tobacco use (yes)	9.64	0.48-18.81	**0.039**
Number of medical co-morbidities	3.48	1.32-5.64	**0.002**
NSAIDs (yes)	2.37	-3.49-8.23	0.427
Biologics (yes)	-2.46	-8.33-3.40	0.409
Erythrocyte sedimentation rate	0.41	0.23-0.59	**< 0.001**
Disease duration	0.37	0.15-0.58	**< 0.001**
BASRI	1.74	0.97-2.50	**< 0.001**
Frequency of exercise/week	-0.19	-1.44-1.06	0.766
Back exercise (yes)	-7	-13.11--0.893	**0.025**
Physical therapy (yes)	8.75	-1.63-19.13	0.098
Resilience coping (BRCS)	-1.42	-2.30--0.55	**0.002**
Arthritis internality	-1.34	-1.81--0.87	**< 0.001**
Arthritis helplessness	2.76	2.18-3.34	**< 0.001**
Depression (PHQ-9)	2.40	1.90-2.91	**< 0.001**
Active coping	-0.49	-1.04-0.07	0.086
Passive coping	1.39	1.04-1.75	**< 0.001**

BASFI = Bath Ankylosing Spondylitis Functional Index; BASRI = Bath Ankylosing Spondylitis Radiographic Index; BRCS = Brief Resilient Coping Scale; PHQ = Patient Health Questionnaire.

**Table 3:** Hierarchical multivariate analysis of demographic, medical and psychological variables in relation to BASFI

Step	Independent variable	Mean difference	R-square (%)*	*P *value+
1	**Demographic variables**		21.0 *	**< 0.001^+^**
	Age	0.54		**< 0.001**
	Employment	-13.58		**< 0.001**
	Gender	-4.68		0.122
	Marital	2.15		0.491
	Education	-3.80		**0.001**
	Student	2.75		0.669
	Ethnicity	-4.38		0.259
				
2	**Medical variables**		32.0*	**< 0.001^+^**
	Current tobacco use	9.12		0.099
	NSAID therapy	6.56		**0.021**
	BASRI (range 1.5 -- 16)	0.75		0.134
	Biologic therapy	4.96		0.052
	Number of medical co-morbidities	2.08		0.19
	Erythrocyte sedimentation rate	0.36		**0.001**
	Disease duration	0.00		0.86
	Days of general exercise per week	-1.5		**0.036**
	Back exercise	-6.35		0.08
	Physical therapy	12.65		**0.024**
				
3	**Psychological variables**		56.3*	**< 0.001^+^**
	Arthritis internality (6-36)	-0.54		**0.048**
	Arthritis helplessness (5-25)	1.14		**0.004**
	Resilience coping (BRCS) (0-20)	0.32		0.507
	Depression (PHQ-9) (0-27)	0.90		**0.013**
	Active coping (7-35)	-0.12		0.703
	Passive coping (11-55)	0.68		**0.006**

*Overall R-square (%) after the addition of each conceptual block. +Overall *P *value after the addition of each block. BASFI = Bath Ankylosing Spondylitis Functional Index; BASRI = Bath Ankylosing Spondylitis Radiographic Index; BRCS = Brief Resilient Coping Scale; PHQ = Patient Health Questionnaire.

**Table 4:** Final model of correlates of the BASFI

Independent variable	Mean difference	95% confidence interval	R2 (%)	*P *value
Overall model			48.8	**< 0.001**
Age (at baseline visit, V0)	0.31	0.11-0.51		**0.002**
Depression (PHQ) (higher score = more depression, range 0-27)	1.20	0.58-1.82		**< 0.001**
Arthritis helplessness (higher score = more helpless behavior, range 5-25)	1.31	0.62-2.01		**< 0.001**
Passive coping (higher score = more passive coping, range 11-55)	0.69	0.24-1.13		**0.003**
BASRI (range 1.5-16)	1.28	0.61-1.95		**< 0.001**
Erythrocyte sedimentation rate	0.33	0.17-0.48		**< 0.001**

BASFI = Bath Ankylosing Spondylitis Functional Index; BASRI = Bath Ankylosing Spondylitis Radiographic Index; PHQ = Patient Health Questionnaire.

## Results

### Sample characteristics

A total of 294 patients were included in the study. Table 1 shows patient demographics, medical, and psychological testing scores. The mean (standard deviation) age of the sample was 45.1 (± 14.40) years, 68% of the cohort was male, and 82% of the sample was white. The mean disease duration at study baseline was 21.23 (± 13.85) years, and less than half of the sample was taking NSAIDs (47%) and/or biologic agents (45%). The majority of patients (64%) were performing back exercise while only a small portion of patients (9%) was undergoing physical therapy for treatment of AS. Participants reported a high level of resilient coping (mean score 16.09 ± 3.33) and relatively low depression scores (mean score 5.14 ± 5.01). The mean score for arthritis internality was 25.66 (± 5.94), for helplessness was 12.42 (± 4.41), for active coping was 22.74 (± 5.52), and for passive coping was 25.59 (± 7.45). The latter scores are all within one standard deviation of mean scores obtained from samples of patients with RA [24,30] on these measures.

### Univariate analyses

The univariate regression analysis found the following variables to be significantly associated with higher BASFI scores (more functional limitation): older age, tobacco use, number of medical co-morbidities, higher ESR, disease duration at baseline visit, higher BASRI scores (more radiographic disease damage), high passive coping, high helplessness, and high depression scores. Low education level, unemployment, low resilience coping and low internality also significantly correlated with higher BASFI scores while performance of back strengthening or stretching exercise was associated with lower BASFI scores The other variables examined, including gender, ethnicity, marital and student status, current use of biologic therapy and NSAIDs, frequency of exercise, treatment by physical therapy and active coping did not significantly correlate with BASFI scores (Table 2).

### Hierarchical modeling with successive conceptual blocks

In order to evaluate the factors contributing variance to BASFI scores, the independent variables were added into the analysis in the following successive conceptual blocks: socio-demographic variables; medical variables; and psychological variables. First, the demographic variables were entered. The contribution of these variables accounted for an overall R-square of 0.21 (*P *< 0.001. Advanced age (*P *< 0.001), unemployment (*P *< 0.001), and low education level (*P *= 0.001) contributed independent variability to BASFI scores. The addition of the medical variables, including current tobacco use, current NSAID and/or biologic therapy, BASRI scores, medical co-morbidities, ESR, and disease duration, frequency of exercise, performance of back exercises and treatment by physical therapy resulted in an R-square of 0.32 (*P *< 0.001). However ESR (*P *= 0.001), NSAID use (*P *= 0.021), frequency of exercise (*P *= 0.036) and treatment by physical therapy (*P *= 0.024) were significantly related to BASFI scores after correction for other demographic and clinical factors. The other variables, including current tobacco use, biologic therapy, disease duration, number of medical co-morbidities, performance of back exercises and radiographic damage scores did not reach statistical significance in the hierarchal model. Finally, the entry of arthritis internality, helplessness, resilient coping, depression, active coping, and passive coping resulted in an R-square of 0.56 (*P *< 0.001). Higher depression (*P *= 0.013), helplessness (*P *= 0.004), passive coping (*P *= 0.006), and lower internality (*P *= 0.048) had significant, independent associations with BASFI scores, while the contribution of active coping (*P *= 0.703), and resilience coping (*P *= 0.507) were not significant. As an aggregate, the psychological variables contributed significantly to the overall variance, adding an additional 24% variance above that accounted for by demographic and medical variables (Table 3).

### Final model

The hierarchical forward model found that higher helplessness (*P *< 0.001), depression (PHQ-9; *P *< 0.001), passive coping (*P *= 0.003), ESR (*P *< 0.001), radiographic severity scores (BASRI) (*P *< 0.001), and older age at baseline visit (*P *= 0.002) were significantly associated with higher BASFI scores (Table 4). These variables explained 49% of variance in BASFI scores. More specifically, each numerical increase (range of scores 0 to 27, with higher numbers equaling more depression) in depression resulted in an increase of 1.20 in the BASFI score (scale score 0 to 100 mm), and each numerical increase in the arthritis helplessness score (range of scores 5 to 25, with higher scores indicating more helpless behavior), resulted in an increase of 1.31 in the BASFI score. Although age, radiographic severity scores, and ESR were significant in the final model, the remaining demographic and medical factors, including number of medical co-morbidities, use of NSAID and biologic therapy, exercise habits and disease duration, failed to explain a significant portion of the variance of BASFI scores in the final model. Inspection of the variance inflation factor did not suggest multicollinearity among predictors in the final model.

## Discussion

Six variables, higher arthritis helplessness, depression, passive coping scores, ESR and radiographic disease scores (BASRI), and older age correlated significantly with more functional limitations in our cohort.

Although prior studies have demonstrated an association between systemic inflammation, radiographic severity, older age and functional limitation, this is the first study to investigate the role of psychological factors beyond the demographic and clinical factors in a multivariate model. The results demonstrated a strong correlation of psychological variables to AS functional limitations. Specifically, higher arthritis helplessness, depression and passive coping correlated significantly with more functional limitation in the final model, mirroring findings in other chronic arthritic conditions [19,24,29,31-34]. Our findings are also consistent with a previous report linking helplessness to worse health-related quality of life in AS [35]. In contrast to passive-coping, active coping did not significantly correlate with loss of functional abilities, a finding which has also been reported in other arthritic conditions, such as RA [36,37]. Passive coping may be a more robust contributor to functional limitation due, in part, to its association with depression and poor psychological functioning, a result found in both rheumatic diseases and in traumatic injuries such as whiplash [38]. Coping researchers have theorized that successful coping is not solely the result of using adaptive coping strategies, but also the absence of the frequent or continuous use of maladaptive strategies [39,40]. In AS, while passive coping may be a common response during acute disease flares, the more that patients rely on passive pain coping on a daily basis, the more difficulty they may encounter in sustaining functioning and quality of life.

Interestingly, when psychological factors were included in the analysis, variables previously found to be significantly associated with functional limitation, including current tobacco use, education level, gender, and number of co-morbid medical conditions, performance of back exercises failed to show significance. These findings indicate that psychological factors accounted for some of the variability in functioning that was originally contributed by these socio-demographic and medical variables. Our data also indicate that psychometric variables should be evaluated and accounted for when assessing functional limitations of AS patients in observational or interventional studies. It also is possible that psychological variables could mediate the effect of important socio-demographic and medical factors on functional limitations in patients with AS. For example, smoking, which has been shown to have a strong association with the progression of functional limitation, could be a surrogate of a psychological health behavior or mood disturbance which, in turn, could affect disability. Further research exploring mediational pathways underlying AS disability would clarify such propositions.

The independent relation between depression and functional limitations in AS is consistent with other literature in rheumatic conditions showing that mood disturbance is a covariate of both disease activity and disability, particularly in RA [41,42]. Due to the cross-sectional design of the current study, it is not possible to discern whether depression is a cause of disability in AS, a result of the impact of the disease, or a product of an underlying inflammatory process. Future research in AS could address these important questions and shed light on the issue of managing depression in affected patients.

Both age and disease duration have been found to influence functional limitation in AS. However, as these variables tend to be collinear, it is difficult to distinguish their individual effects. Although age was significantly correlated with functional limitations in this study, disease duration was not, indicating that age had an influence on functional limitation, apart from its association with duration of disease. It could also be presumed that the number of co-morbid medical conditions could influence the association of age with functional limitation. However, in this analysis, age was significantly correlated with functional limitation, while co-morbid medical conditions did not achieve significance. The importance of age, independent of its association with disease duration or potential contribution of co-morbid medical conditions, has been noted previously [43].

The primary limitation of the present study was the cross-sectional study design, which provided only correlational findings, precluding an understanding of directional relations among model variables. For example, it cannot be determined from our data whether higher helplessness, depression, and passive coping scores caused a heightened perception of functional limitation or vice versa. Another point to consider is the possibility that patients with higher depression scores over-report functional limitation (i.e. reporting bias). A longitudinal study, in which patients' psychological status and functional limitation are monitored over time, is needed to determine directionality. It is conceivable that there is a bidirectional relation between the perceived functional limitation and psychometric factors. Furthermore, the observed strong correlation between BASFI and the utilized psychometric instruments might be partly explained by the fact that these scales are all based on patient reports. It is important that these types of studies are extended to more objective scales of physical limitation such as the Bath AS Metrology Index [44]. Furthermore, a smaller portion of our patients was treated with NSAIDs than in European cohorts, which might affect the generalizability of the observation that this therapy modality was not associated with higher functionality in AS [45]. Also, our study cohort was primarily white and well educated, which could limit the generalizability of our findings to other socioeconomic or ethnic groups. As the level of work disability and functional limitation has been shown to vary inversely with formal education level [5,7,13], the results might have varied with a group possessing a wider range of education levels.

## Conclusions

Our findings support those from prior studies, showing a strong association of older age, radiographic severity, and elevated inflammatory markers with functional limitation in patients with AS. However, we also report novel findings showing a strong correlation of psychological variables, specifically arthritis helplessness, passive coping, and depression, with functional limitations beyond the effect of the known clinical and demographic variables. These results have implications for clinical practice, because interventions that focus on our patients' psychological health may be another mechanism to slow the rate of perceived functional decline in the AS population.

## Abbreviations

AHI: Arthritis Helplessness Index; AS: ankylosing spondylitis; BASDAI: Bath AS Disease Activity Index; BASFI: Bath AS Functional Index; BASRI: Bath AS Radiographic Index; BRSC: Brief Resilient Coping Scale; ESR: erythrocyte sedimentation rate; NSAID: non-steroidal anti-inflammatory drug; PHQ-9: Patient Health Questionnaire-9; PSOAS: Prospective Study of Outcomes in Ankylosing Spondylitis; RA: rheumatoid arthritis; VAS: visual analogue scale; VPMI: Vanderbilt Pain Management Inventory.

## Competing interests

The authors declare that they have no competing interests.

## Authors' contributions

TFB, SA, JDR, TL, LD, MHW, JCD, MHW and PN contributed to the study design, data acquisition and drafting of the manuscript. Furthermore, TFB, SA, MHW and PN contributed substantially to data analysis and interpretation. All authors approved the final version of the manuscript to be published. All the pelvic radiographs were reviewed and scored by TL.
